# Supplementary material for: Awareness and practice of medical waste management among healthcare providers in National Referral Hospital
Source: PLoS One. 2021 Jan 6;16(1):e0243817. doi: 10.1371/journal.pone.0243817 (PMC7787467; doi:10.1371/journal.pone.0243817)
Supplement: S1 File — (DOCX) [file pone.0243817.s001.docx]

**The Demographic Questionnaire**

**Direction:** Please fill your information’s below.

1. Gender

Male Female

1. Age ……………Years
2. Level of education

Certificate

Diploma

Bachelor’s degree

Higher than Bachelor’s degree

Others

1. Years of experience ……….Years...……… Months
2. What is your current place of work/area of specialty?

Emergency Room Pharmacy

Medical/Surgical Critical Care

Pediatrics Dialysis

Oncology Obstetrics/Gynecology

Laboratory Others

Please specify…………………………………

1. What is your profession/designation?

……………………………………………………..

1. Have you ever attended training or workshop concerning waste management?

Yes No

1. If yes to question No. 7,

Please specify…….……………………………………………….

1. How many needle stick injuries reported in the past 12 months?

……………………….

1. Did you vaccinate yourself against hepatitis B and tetanus?

Yes No
