# Supplementary material for: Awareness and practice of medical waste management among healthcare providers in National Referral Hospital
Source: PLoS One. 2021 Jan 6;16(1):e0243817. doi: 10.1371/journal.pone.0243817 (PMC7787467; doi:10.1371/journal.pone.0243817)
Supplement: S2 File — (DOCX) [file pone.0243817.s002.docx]

**PART II: AWARENESS QUESTIONNAIRE**

**Direction:** The response will be coded by enumerators based on response given by participants on face to face interview.

| **Parameters** | **Aware** | **Not Aware** |
| --- | --- | --- |
| Have you ever heard about medical waste |  |  |
| Are you aware of regulation on medical waste management |  |  |
| Do you know about the biohazard symbol |  |  |
| Can you name eight categories of medical waste |  |  |
| Can you list down the guidelines provided for color coding in workplace |  |  |
| What is puncture‑proof container for sharps |  |  |
| What is the correct bag for disposal of cytotoxic drugs |  |  |
| What is the correct bag for intravenous sets, catheters, and tubes |  |  |
| HIV/AIDS can be transmitted through medical waste |  |  |
| Hepatitis B and C can be transmitted through medical waste |  |  |
| Personal protective measures are necessary while handling medical waste |  |  |
| When do you discard medical waste from the bin |  |  |
| Do you know about methods for medical waste treatment |  |  |
| Disinfection of medical waste is necessary |  |  |
| Bleaching solution 0.5% is used for disinfection of infectious medical waste |  |  |
| The maximum time for medical waste to be kept in hospital premises is 48 hours |  |  |
