# Supplementary material for: Awareness and practice of medical waste management among healthcare providers in National Referral Hospital
Source: PLoS One. 2021 Jan 6;16(1):e0243817. doi: 10.1371/journal.pone.0243817 (PMC7787467; doi:10.1371/journal.pone.0243817)
Supplement: S3 File — (DOCX) [file pone.0243817.s003.docx]

**PART III: OBSERVATION CHECKLIST FOR MEDICAL WASTE MANAGEMENT**

**Direction:** Please fill up as indicated based on observation.

Name of the ward/unit:

Participant: **Observation**

| **Parameters** | **Yes** | **No** | **NA** |
| --- | --- | --- | --- |
| ***[ON – SITE OBSERVATION]***  **A. Condition of waste receptacles** |  |  |  |
| Is green colored waste bin available in ward |  |  |  |
| Is yellow colored waste bin available in ward |  |  |  |
| Is red colored waste bin available in ward |  |  |  |
| Is blue colored waste bin available in ward? |  |  |  |
| Has green bag been placed lining the inner side of green bin? |  |  |  |
| Has red bag been placed lining the inner side of red bin? |  |  |  |
| Has blue bag been placed lining the inner side of blue bin? |  |  |  |
| Is green bag securely fitted with the bin |  |  |  |
| Is red bag securely fitted with the bin |  |  |  |
| Is blue bag securely fitted with the bin |  |  |  |
| Are waste bins covered |  |  |  |
| If covered, is cover foot-operated |  |  |  |
| Is the biohazard symbol imprinted over waste bags |  |  |  |
| Are posters to guide users displayed near waste bins |  |  |  |
| **B. Segregation of waste** |  |  |  |
| Does green bag contain only general waste |  |  |  |
| Does yellow bag contain only sharp waste |  |  |  |
| Does red bag contain only soiled infected waste |  |  |  |
| Does blue bag contain only food waste |  |  |  |
| **C. Transportation of medical waste** |  |  |  |
| Appropriate on-site transport of medical waste used |  |  |  |
| Is transportation of medical waste done during non-busy hours |  |  |  |
| Are infectious and general waste transported separately |  |  |  |
| **D. Appropriate use of PPE** |  |  |  |
| ***[OFF – SITE OBSERVATION]***  **E. Mutilation of recyclable waste** |  |  |  |
| Are used hypodermic needles destroyed |  |  |  |
| Is nozzle of used syringes destroyed |  |  |  |
| Are used hypodermic needles found re-capped |  |  |  |
| Are used hypodermic needles found bent |  |  |  |
| Are used plastic bottles cut |  |  |  |
| Are used plastic tubing’s cut |  |  |  |
| **F. Segregation and Disinfection** |  |  |  |
| Are general and infectious waste bags segregated |  |  |  |
| Sharp wastes stored/secured properly |  |  |  |
| Pathological waste disposed in deep burial pit |  |  |  |
| Cytotoxic waste stored separately |  |  |  |
| Is waste generated recorded in treatment site |  |  |  |
| Are transportation trolleys/vehicle disinfected after disposal |  |  |  |
| **F. Appropriate use of PPE** |  |  |  |
