# Supplementary material for: Awareness and practice of medical waste management among healthcare providers in National Referral Hospital
Source: PLoS One. 2021 Jan 6;16(1):e0243817. doi: 10.1371/journal.pone.0243817 (PMC7787467; doi:10.1371/journal.pone.0243817)
Supplement: Attachment — Submitted filename: Response to reviewers.docx [file pone.0243817.s005.docx]

Review and Recommendations PONE-D-20-06712: **Awareness and Practice of Biomedical Waste Management among Healthcare Providers in National Referral Hospital**

**Research Summary:**

The authors conducted an observational cross-sectional study to evaluate healthcare provider awareness, knowledge, and practice concerning medical waste handling at a national referral hospital. The study sought to assess the familiarity with- and practices used by a variety of healthcare workers in their current work at a large medical facility as it pertains to their daily work assignments involving the handling of medical waste generated during patient care. While study participants were aware of potential risks associated with handling and disposing of medical waste, only a small proportion of the them followed best waste management practices. National and international guidelines exist regarding the proper handling of medical waste and the identification of potential health risks to healthcare providers improper handling presents. Deficiencies contributing to challenges encountered in the proper handling and full implementation of a compliant medical waste handling program are identified.

The identification of specifics regarding deficiencies and discussion of their potential impact is limited. The manuscript would be enhanced by providing additional descriptive information about training programs, facility enforcement measures, the range of available personal protective equipment and its use by the healthcare providers as well as what program resources are dedicated to medical waste program activities. Background information introduced in different sections of the manuscript could be pulled together more completely earlier in the document. Additional description in the methods of the rationale for approaches used in this study would enhance understanding of how the authors selected the evaluation tools used. This would add additional insight into any challenges investigators encountered. Including the strengths and limitations of the study and the methods used will provide other investigators with additional considerations for further research efforts involving medical waste handling programs in healthcare settings. Inclusion of some overall findings from this study to support the authors’ conclusion and recommendations would provide readers seeking considerations for improving medical waste handling in healthcare settings and the protection of healthcare workers from potential hazards associated with medical waste is suggested.

Response: National guidelines on infection control and waste management is used and in JDWNRH currently, inspection on infection control is carried out bi-annually (report attached)

**General Reviewer comments:**

From my review of the manuscript it appears that the investigators’ presentation of their findings would benefit from some additional revision. The current organization of the manuscript along with identified information gaps suggest additional work is needed to refine this work prior to publication and reflect more fully the extensive work that was done. More in-depth examination of the data collected along with additional background and more extensive discussion of their findings would help develop their findings and the potential value of this work for other researchers and readers interested in this topic. While all of the data (responses) from the study participants that is presented in the manuscript appears to be found in the tables, I think the results and their presentation as well as the discussion and conclusions would be more informative if additional findings extracted from the collected data across respondents would have been included. Greater characterization within the subgroups of their attributes and then comparison with the other groups within the larger category could provide additional insights strengthening your findings. More specific comments and questions are provided throughout this review. The authors may want to consider whether incorporating some graphical presentation of their results would help with conveying the results rather than leaving it only to some large tables backing up the written results presentation. Were any photos obtained as part of the observational checklist process? That could add to the presentation of the findings. The manuscript organization along with the varying use of subheadings became increasingly confusing from lines 102 – 199. Please spell out acronyms the first time they are used.

Response: Following other references from the PLOS one publications, the tables were found appropriate in disseminating the information’s and all the authors agreed on keeping the results in tables. Additional pictures from the observation checklist is added

**Review Comments, Questions, and Suggestions:**

**Title and Abstract:**

The type of study is presented in the Methods paragraph of abstract. In the Objectives study aims are identified but only include assessment of awareness, not practice which is indicated in the title. The sentence on lines 27-28 could be clearer. Is it that 74.4% of the study participants are aware of medical waste management and that 98.2% of those individuals use personal protective measures (or equipment)? The sentence on lines 29-30 is not clear. Is it 61.3% of the observed study participants correctly segregate the waste according to national guidelines – but that less than half of the facility’s waste is apparently segregated? It is not clear where this figure comes from in this study or how to interpret it in this sentence. In the second Conclusion sentence (Line 34) does this recommendation apply to this study’s findings or is this affirming something more broadly encountered? The conclusion statements appear to be more broadly applied than the actual study appears to have addressed since it looked at one facility over a fairly short period of time. Study participant selection used a convenience sample with no explanation supporting this choice. This work, while it may reinforce the findings from other studies regarding the need to address deficiencies found at this facility, could benefit from adding qualifiers acknowledging study limitations. Consider including the term “training” among the Keywords since this appears to be an

Response: Practice has been included. The lines not clear has been deleted. Convenience sampling was chosen in order to target all the healthcare personnel who works in shift-system at JDWNRH. JDWNRH being the apex hospital with 350-beds is serves the larger number of population with higher wastes being generated.

**Introduction:**

While the introduction section is quite long and includes a lot of background for the study, additional background information is found later throughout the manuscript. Consider identifying both an Introduction and a Background section, providing the reader with the background information in one place. This will then give the reader informative background material that frames the next sections of the manuscript and provides a more concise justification for the work that is subsequently described. Provide more specificity in the background material to tighten up the concerns supporting this work.

Lines 39-40 address contamination of “ground” water from landfills but could be more specifically stated by giving some examples regarding what it may become contaminated with from these wastes. Hazardous biomedical waste may include microorganisms (which includes viruses, bacteria, fungi, prions), active pharmaceutical ingredients and their metabolites, excreta containing radioisotopes, contaminated sharps, etc.

Appropriate medical waste management in line 47 does not include collection yet this is mentioned elsewhere in the manuscript and for completeness should be included here. Please specify what “it” refers to at the end of line 50. More complete description of the storage containers on line 51 with their characteristics would be informative regarding concerns mentioned later with handling the collected waste. Are the containers puncture resistant, placed inside of a second outer container, of a minimum specified thickness of material, and do they use a secure closure system to reduce unintentional release of contents? Line 51 mentions a 72-hour time limit yet later it is mentioned that medical waste is to be held on-site no longer than 48 hours. What are examples of improper storage that were encountered and how did the lack of human resources often contribute to the observed problems? Provide an indication of the size of the facility studied in the study cited in reference 8. Does it have similarities or differences with the facility this study evaluated? Provide a more specific citation for reference 9 as to whether this is a government document, contractor or agency report, etc. that would assist a reader that wished to locate this document.

Lines 55-59: be more specific as to what medical wastes are processed and how prior to final disposal. This is a bit confusing. Untreated or partially treated medical waste also may present potential hazards to workers handling it downstream from the point of generation at the medical facility prior to its placement in the final disposal site.

Lines 67-68: provide background information describing the protective gear (personal protective equipment) that is available to- and used by- the workers. Include if this varies by worker groups and locations.

Line 68-70: suggest including reference number 24 by Yong-Chul et al. which is cited in your reference 3 pertaining to medical waste challenges encountered in Korea. That appears to be the reference applicable to the mention of Korea’s experience.

Line 74-75: is there a missing reference here? A governmental act of 2009 is referred to but both reference 5 and 10 are dated well after the mentioned act.

Lines 80-89: It is unclear here what is meant by segregation of medical waste into two categories – on-site and off-site. Is the segregation based upon where the waste is generated, is it segregated at the waste treatment site, are hazardous and non-hazardous medical waste comingled with additional processing further down the waste management stream? You appear to be describing a problem here with medical waste handling but it isn’t clear what the problem is. This is important background in helping define the problem(s) you are evaluating. Mention of on-site and off-site medical waste management and workers appears in lines 134-135 where it is also unclear what this means.

Lines 97-100: Additional information that belongs in background.

Response: Additional information from the guidelines are cited to provide clear information on the waste collection, segregation, and transportation and disposal method. Changes in references cited made.

**Research Objectives:**

Please elaborate. Are there groups, job titles, facility components, patient care delivery locations, job titles or some specific issues that are a concern the objectives are to address? This builds on the background. Currently while this is very brief it is also extremely broad. Use the background to lead into the objectives. This will help frame the context of your following study.

**Methods and Materials:**

**Study Design:**

Line 100: A reference is needed for this Annual Report citation. This and the next line appear to define the study cohort. This would benefit from some further elaboration of what health workers are included and possibly what the specific selection criteria were. Inclusion criteria appear in lines 116-117, but it still needs some further elaboration. Are these only permanent full-time facility employees versus contractors, volunteers, and all the people falling under lines 118-119 Exclusion Criteria? Does paramedics include those employed by the hospital as well as those employed by private or other governmental entities?

Response: Only full time employees were interviewed. And no inclusion from any private organizations. The staff working in various department as listed below were used for the study:

1. Department of Anesthesiology and Critical Care
2. Department of Community Health
3. Department of Dentistry
4. Department of Dermatology
5. Department of Emergency Medicine
6. Department of Forensic Medicine
7. Department of Medicine
8. Department of Obstetrics and Gynecology
9. Department of Ophthalmology
10. Department of ENT
11. Department of Orthopedics
12. Department of Pediatrics
13. Department of Pathology and laboratory Medicine
14. Department of Physiotherapy
15. Department of Pharmacy
16. Department of psychiatry
17. Department of Radio Diagnosis and Imaging
18. Department of Surgery

**Sampling, Sample size:**

Lines 103-114: This should be developed further. What is the rationale for the selection of a convenience sampling method? What limitations or logistical considerations supported this decision? Did this approach and its potential limitations offset potential issues of using some other approach? Were there any additional actions taken to address the limitations this brings to the study to attempt to offset the limitations? Were there feasibility considerations that argued for this approach and what were they? Please identify the 18 departments or units of JDWNRH that were included in this study and why they were selected.

Response: convenience sampling method was used to get as many respondents as possible for this study since the staff works in shift-system.

**Sample size:**

A citation in the references is need for this. This section would benefit from some additional explanation of how you arrived at the values used for the variables used in your sample size formula to obtain final sample size. Explain if there were any additional assumptions that had to be used. How were study subjects recruited? What does line 114 mean about the need “…to cover up the missing data.”? No discussion is provided for the observational checklist portion of the study.

Response: Reference cited Observation included checking the dustbins at the wards/departments etc as mentioned in supporting document based on observation checklist and visiting the treatment site to check the procedure for treatment and disposal process

**Inclusion criteria:**

Previously addressed under study design. Is one correct to assume this also excludes individuals who may be foreign nationals, permanent non-citizen residents, guest researchers, non-employee consulting experts, and visiting scientists? It may be appropriate to provide the definition of a Bhutanese citizen as well as who is included in the domain of health workers.

Response: Yes

**Research instruments:**

This study used three data collection instruments to gather data from participants. No information is provided concerning how these instruments were developed. No indication is provided as to whether the data collection instruments drew upon past collections of questions or survey instruments. There is no indication of whether the survey instruments were pilot tested prior to their final use for data collection or whether any bank of standardized questions was drawn upon in assembling the questionnaires. How were the three questionnaires for individual participants linked together for data analyses? It did not become clear to me until reviewing the results table for the observational checklist that it is a separate assessment from the first two evaluation instruments. What was learned from the first part of the study is not directly connected to this last portion.

Response: The research instrument was developed based on WHO guidelines (2014) and were pilot tested prior to dissemination to the participants.

**Demographic Questionnaire:**

Was it the intention to ask respondents about self-vaccination for Hepatitis B and tetanus in the last question or if they were currently vaccinated against these two illnesses?

Response: Yes

**Awareness Questionnaire:**

This survey instrument contains 15 questions rather than questionnaires (line 129). Contrary to previous administration description in lines 122-124, this instrument was stated in line 130 to have been completed with a face-to-face interview. This questionnaire used a yes-no response format. Please elaborate on what a correct or incorrect response is for questions that are answered “yes” or “no” when asking whether the individual does or doesn’t know something.

Response: If an individual does know something was considered as ‘yes’ and doesn’t know as ‘no’

**Observational Checklist:**

Departments referred to in line 135 are not presented above as is parenthetically indicated. The mention of on-site and off-site among health care providers appears again. It is unclear what this means. The checklist appears to be an assessment of process, work practice, and handling procedures separate from the health care providers individual knowledge and practices evaluated by the first two instruments.

Response: The observational checklist provided a real-time assessment of the waste management by visiting their work site. The other two questionnaire checked the knowledge of the healthcare workers whereas the observational provided the practice of waste management

**Data collection procedure:**

Confusion exists about the completion of these instruments. They were reported to be all self-reports and checklists completed by the study participants (lines 122-124). Later under Data Collection (lines 141-142) there is mention that the information was collected by face to face interviews. It is also unclear under Data Collection Procedure, line 140, what if anything was explained to the participants versus department and unit supervisors (“in-charges”).

Authors do not indicate what information was conveyed to the actual participants concerning this study and their participation, the data collection process, the use of- and access to- the collected data, the consent process for participants, whether participation was voluntary or mandatory, any consequences associated with non-participation or participation withdrawal, and whether participation was incentivized.

Response: The participants consent were obtained prior to interviewing and all the Head/In-charges were notified about the study. All the participants were voluntary.

**Data analysis:**

Provide references for the data management and statistical software programs. Were there any issues with- or how were any missing data points handled? Line 166 shows a total participant N of 340, whereas line 114 under Sample Size presents an N of 350. This should be reconciled.

**Results:**

This section is the results presentation for the manuscript. It relies heavily on the tables with brief text description of what is in the tables. Line 152 entitled Part 1: Awareness about Biomedical Waste Management is followed by the results from each of the three data collection tools. There is no Part 2 or 3. Consider renaming this section Results with a more complete text presentation. Provide the three tables following the references at the end of the manuscript. The use of tables should not replace a coherent text presentation of the findings. The authors may want to consider using some graphic presentations of their most salient findings and allow the reader to review the tables of the results at the end of the manuscript.

Response: Changes made in the results section

**Demographic characteristics of participants and Table 1**

Line 157 indicates that the age range of most health care providers was between 26 and 30 years of age, yet the mean age was 32.2 years. While the 26-30 year age group may be the largest single age group of the four age categories identified, it makes up only 33.8% of the study population. The second largest age group is over 35 years of age. A more complete presentation of what was found would be informative. The second and third largest groups are both older than the single age group presented.

What does Others encompass for level of education? It is twice the size of the smallest category for the Level of Education attribute.

The largest group as far as Place of Work is Others. Explain reasoning for lumping together such a large group. Table 1 provides the first presentation of a listing of where participants worked. This should be presented in an earlier section of this manuscript, possibly Background or in describing the study population.

Profession also has as its third largest group Others. Table 1 is also the first place a listing to any extent of the job titles included in the study population appears. This should be presented earlier, possibly in the Inclusion Criteria for the study population.

Response: Since ‘others’ consist of 8^th^ and 10^th^ grade passed employee who works as cleaners at the hospital. The participants also involved those who worked closely in disposing the wastes from the wards/departments. Therefore, they were clumped under ‘others’

**Awareness of medical waste management among health care providers (not identified as the next results discussion) and Table 2**

Where does the overall value of 74.4% awareness of medical waste management among health care providers come from in the Table 2 data? This number and the percent totally unaware presented in lines 169-170 coincide with the awareness question about recognizing the biohazard symbol. That doesn’t seem equivalent to awareness or not of medical waste management, unless awareness is considered recognition of a marking symbol. Some additional definition of awareness for the purposes of defining a correct or incorrect response may be helpful back under the Awareness Questionnaire description, at lines 129-132.

Line 172 appears to present the same data from line 169 for another attribute. For line 173, is the 80% referring to a fraction of the 45.3% in the previous line? In line 174, is it supposed to be the correct bag “color” for disposal of cytotoxic drugs or is it a totally different bag unlike that used for anything else?

Line 177 mentions personal protective measures, but these measures are not well defined anywhere in the manuscript. That would be something else to consider including in the Background Section.

Response: Changes made and rectified

**Observational checklist results (not separately identified) and Table 3**

It does not become evident until more closely reviewing the results in Table 3 that the observation checklist does not necessarily have any direct link with the study participants work practices or knowledge presented in Tables 1 and 2. It appears from Table 3 that 31 observations were made. There is no mention of how this number of observations was determined. The departments evaluated are not mentioned anywhere previously nor are they identified here. There is no indication as to the sampling strategy for selecting the 31 observation locations or how representative they are of the various medical waste collection and handling activities that occur at this facility. Availability and types of available personal protective equipment is first mentioned in lines 230-234, just before the Conclusion.

Response: Changes made and rectified

**Discussion:**

Line 203-204 more appropriately belongs in the Methods section. Much of the discussion appears to be a recap of the results.

Lines 209-211: Please explain why this finding is considered noteworthy. Lines 211-215 seem quite nebulous. What about within-facility medical waste program auditing and work practice reinforcement? What role does the facility’s worker health and safety or environmental health and safety program have or is this something needing to be further addressed? What resources are available for and where does the responsibility lie for implementing, auditing, and reinforcing proper medical waste handling procedures? Would some recommendations be appropriate to improve the situation identified regarding the medical waste program based upon these study findings?

Line 219: The reference cited here refers to the National Integrated Solid Waste Management Strategy, National Environment Commission, Thimphu Bhutan 2014. Is this correct since the intended reference appears to be for a medical waste study conducted in Nigeria?

Please explain the sentence in lines 219-220. How does color coding of most waste bins indicate understanding of the respondents of the management of medical waste? Do the respondents have the responsibility for color coding the bins? Does the waste stream placed into the bins match the types of waste the color coding indicates should be placed into it?

Lines 224-226: Please elaborate how and why this could be dangerous. The subsequent sentences suggest that a comingling of hazardous medical waste and non-hazardous waste may be occurring which could render all the wastes handled potentially hazardous. Is this something that the study identified occurs at this facility, and if so, this didn’t seem to come out in the results.

Identification of the strengths and limitations of this study should be added.

Response: Changes made and rectified

**Conclusion:**

Investigators should make sure that what is covered in the conclusion has been developed in the preceding discussion.

**Declarations:**

No comments

**References:**

References should be complete to provide the interested reader with enough information enabling them to obtain the cited document or publication. For government or contract report documents this may include agency, program, city, country, official publication number, date, number of pages, contract number, contractor preparing report, etc. depending upon required reference format. Several citations are given in the manuscript and do not show up in the references. These are found on lines: 75, 100,106, 146. There is also a secondary reference (reference 24) that is found in the *BMC Public Health* article cited as reference number 3 in this manuscript and should be added at the end of line 70.
